# PteroBot: A Forest Exploration Robot Bioinspired by Pteromyini Gliding Mechanism

**DOI:** 10.3390/biomimetics10100661

**Published:** 2025-10-01

**Authors:** Minghao Fan, Jiayi Wang, Tianyi Liu, Ze Ren, Guoniu Zhu, Jin Ma

**Affiliations:** 1 School of Automation and Intelligent Sensing, Shanghai Jiao Tong University, Shanghai 200240, China; fan_mh@sjtu.edu.cn (M.F.); wjyi117@sjtu.edu.cn (J.W.); tory2004@sjtu.edu.cn (T.L.); joeyr2@sjtu.edu.cn (Z.R.); 2 College of Intelligent Robotics and Advanced Manufacturing, Fudan University, Shanghai 200433, China; guoniu_zhu@fudan.edu.cn

**Keywords:** bioinspired robotics, gliding attitude control, multimodal perception, artificial intelligence, forest exploration

## Abstract

Forests are critical ecosystems that play a fundamental role in supporting biodiversity and maintaining climate stability. However, forest monitoring and exploration present huge challenges due to the vast scale and complex terrain. This paper proposes a novel bionic robot, PteroBot, designed to support a new paradigm for forest exploration inspired by the locomotion of Pteromyini. PteroBot is capable of regulating its gliding posture via a flexible membrane, enabling low-energy and low-disturbance mobility within forest environments. An adaptive gliding control system tailored to the robot’s structure is developed and its effectiveness is validated through aerodynamic analysis, simulation, and experimental testing. Results show that under a cascaded closed-loop attitude controller, PteroBot achieves an average glide ratio of 2.02 and demonstrates controllable turning via attitude modulation. Additionally, comparative tests with UAVs demonstrate that PteroBot offers significant advantages in energy efficiency and acoustic disturbance. Experimental outcomes confirm that PteroBot offers a biologically inspired and ecologically compatible solution for forest exploration, with strong potential in applications such as environmental monitoring, habitat assessment, and covert reconnaissance.

## 1. Introduction

As human activities and the exploitation of Earth’s natural resources continue to intensify, global climate change and ecosystem degradation are accelerating at an alarming rate [1]. Studies have shown that the health of global ecosystems has declined exponentially over the past five decades, with more than 70% of biodiversity facing extinction [2,3,4]. Against this backdrop, the protection and restoration of ecosystems have become an urgent global priority. Fundamental to these efforts is the in-depth exploration of natural environments and the systematic collection of ecological data [5]. However, many critical ecological regions remain largely unexplored, particularly vast forested areas with complex terrain, which serve as habitats for approximately 80% of terrestrial biodiversity [6,7,8].

Currently, forest exploration technologies can be broadly categorized into non-contact and contact-based approaches. Non-contact exploration primarily relies on remote sensing techniques. These systems enable long-term and large-scale monitoring of forest environments. However, remote sensing is limited to observations above the canopy [9]. It also lacks real-time responsiveness and cannot meet the requirements of urgent or dynamic tasks. The other category is contact-based exploration, which allows access beneath the canopy for more detailed data acquisition. This typically requires teams of trained professionals to conduct manual surveys. However, high labor costs and low efficiency make it unsuitable for large-scale exploration. Moreover, the complex and uncharted nature of forest environments poses substantial safety risks to personnel engaged in on-the-ground exploration tasks [10].

To improve efficiency and safety, robotic systems have been increasingly deployed to replace manual efforts in contact-based forest exploration and data collection [11,12,13]. Equipped with onboard sensor suites, these robots are capable of entering regions that are otherwise inaccessible or hazardous to humans, enabling comprehensive environmental observation. Nevertheless, robotic forest exploration presents several systematic challenges. First, terrain complexity means that natural forest terrain exhibits high complexity, being characterized by uneven surfaces, dense undergrowth, fallen branches and leaf litter. Conventional locomotion strategies such as wheeled or legged systems often struggle to maintain stability and mobility under such conditions [14,15]. Second, the spatial constraints of forest gap structure mean that despite avoiding ground obstacles, rotorcraft drones risk collisions in the confined sub-canopy due to cluttered environments of trees, branches, and foliage [16]. Third, endurance and ecological compatibility further constraint drone-based exploration due to limited battery life, which restricts mission duration [17]. Moreover, the loud rotor noise generated by drones can interfere with the natural activities of forest inhabitants, which remains a significant barrier to their widespread deployment in forest environments [18,19,20,21]. These challenges underscore the demand for novel locomotion strategies in forest exploration that are better adapted to canopy gap structures while offering sustained operational capability and ecological compatibility.

Through billions of years of evolutionary processes, life forms have been selected by nature, leading to the development of unique mechanisms finely tuned to their habitats [22]. Inspired by these biological mechanisms, bioinspired robots can enhance their locomotion capabilities in specific environments by mimicking the intrinsic dynamics of biological movements [23,24,25,26]. Moreover, the biomimetic attributes of these robots make it possible for them to operate near local flora and fauna without disrupting the natural state of the environment [27].

This paper proposes an eco-compatible forest exploration robot system named PteroBot that is inspired by the gliding mechanism of Pteromyini. The proposed robot is capable of gliding over complex terrain obstacles and performing forest exploration tasks with minimal ecological disturbance. We analyzed, modeled, and simulated the gliding motion and control of the system. Experiments were then conducted to preliminarily validate its environmentally friendly suitability for forest exploration. The main contributions of this paper are summarized as follows:

1. A novel forest exploration paradigm inspired by the locomotion strategy of Pteromyini is proposed. It pioneers the application of biological gliding mechanisms to robotic forest exploration. This approach leverages the existing forest structure, allowing the robot to glide between densely spaced trees. By avoiding direct interaction with complex terrain and obstacles, it enables efficient and adaptive environmental surveying.
2. Our bioinspired robot called PteroBot is designed to implement the proposed forest exploration paradigm. The robot incorporates structural features of Pteromyini, including deformable wing membranes spanning its limbs along with a broad and elongated tail. Through dynamic modeling, simulation, and experimental validation, the design demonstrates favorable gliding aerodynamic properties. The robot achieves a glide ratio of up to 2.02, approaching the gliding performance observed in Pteromyini.
3. A cascaded closed-loop attitude controller is developed to meet the high responsiveness and stability requirements of gliding. The outer loop is responsible for attitude regulation, while the inner loop controls angular velocity. This control framework simplifies the complex dynamics of low-speed gliding and significantly enhances flight stability. In both gliding and turning simulations and physical experiments, the controller validates its effectiveness by demonstrating rapid and stable responses without overshoot or oscillation.
4. PteroBot has been evaluated in terms of acoustic disturbance, energy consumption, and endurance. The results indicate that the system minimizes ecological disruption to the greatest extent possible. It demonstrates significant advantages in tasks such as wildlife monitoring, environmental conservation, and reconnaissance in ecologically sensitive areas.

## 2. Materials and Methods

### 2.1. Novel Paradigm of Forest Exploration Bioinspired by Pteromyini

#### 2.1.1. Locomotion and Manipulation Capabilities

**Figure 1:**
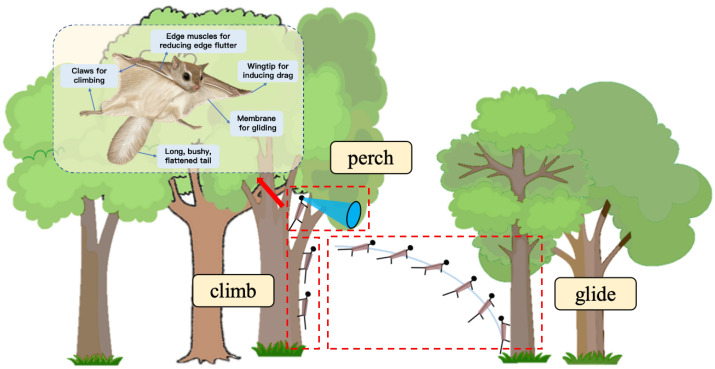
A novel paradigm of forest exploration bioinspired by Pteromyini.

Pteromyini, a rodent species inhabiting the forest canopy, has evolved a unique gliding mechanism in response to the complex forest environment and predation pressures [28]. This mechanism allows Pteromyini to achieve efficient spatial movement with low energy expenditure, enabling it to navigate the complex structures of the forest [29]. Inspired by this locomotion strategy, we propose an innovative forest exploration paradigm. As shown in Figure 1, the robot climbs the tree trunk using its own attachment and climbing capabilities. When a suitable height is reached, it enters a perch mode, stabilizing itself on the tree trunk, and activates multimodal sensors for static environmental perception. After data collection, the robot transitions into a biomimetic gliding phase, which allows it to cross forest gaps with high maneuverability and low energy consumption, moving toward the next target tree. This “climb–perch–glide” exploration paradigm enables the robot to perform efficient and continuous exploration within the complex forest environment.

In this exploration paradigm, controllable gliding is the key to sustained exploration. The gliding aerodynamics of Pteromyini primarily depend on its elastic patagium, a membrane that extends from the forelimbs to the hindlimbs. During gliding, the patagium fully extends to maximize wind-facing area, delaying stall and improving the lift-to-drag ratio (L/D) [30,31]. By asymmetrically contracting the platysma muscle, Pteromyini adjust the orientation of the wing membrane relative to airflow, generating aerodynamic asymmetry along the lateral axis [32]. This adjustment enables Pteromyini to generate a lateral velocity component, facilitating turning maneuvers [33]. Additionally, the flattened tail of Pteromyini provides a pitch attitude control mechanism, assisting in deceleration and stabilizing landings [34].

Recent studies have made notable progress in the bioinspired investigation and mechanical replication of Pteromyini’s gliding mechanism. For example, Li et al. [35,36] introduced the Wave Gliding Gait (WGG) model based on high-frequency, low-amplitude limb movements and performed a dynamic analysis of its aerial posture adjustment. However, this attitude adjustment mechanism focuses mainly on fine-tuning posture stability and does not yet enable further control. To explore more advanced attitude control during gliding, Wang et al. [37] modeled the pitch regulation mechanism of Pteromyini and developed a model for pitch control. Shin and Park et al. [38,39] introduced a walking motion mode in addition to gliding, but their research on gliding posture control remained limited to pitch adjustment. Without further control over the gliding trajectory, obstacle avoidance, and safe arrival at targets, simply increasing the glide distance is not sufficient to optimize exploration in complex environments.

Therefore, turning control during gliding becomes a critical challenge for optimizing gliding trajectories. In this study, PteroBot adjusts its gliding posture through a dual-degree-of-freedom limb mechanism, thereby altering the robot’s lateral aerodynamic characteristics and enabling turning control for maneuverability. This control mechanism not only allows PteroBot to achieve longer glides but also enables effective obstacle avoidance and safe target arrival in complex forest environments, laying the foundation for a sustainable new paradigm in forest exploration.

#### 2.1.2. Perception and Intelligence Capabilities

In addition to basic capabilities in locomotion and manipulation, effective forest exploration requires strong perception and decision-making competence. Existing forest missions often rely on task-specific sensing configurations. For example, thermal cameras are deployed for fire detection [40], while synthetic aperture radar is used in search-and-rescue operations [41]. The exploration approach proposed in this work emphasizes minimal environmental disturbance and high concealment, making it particularly suitable for long-duration tracking and monitoring of specific targets in natural forest settings.

To support these operations, the robot must first achieve accurate self-perception. This is accomplished using an Inertial Measurement Unit (IMU) and joint encoders. The IMU provides data on linear acceleration and angular velocity, while the encoders monitor limb joint positions. Actuators also offer torque feedback, which can help to identify contact points between limbs and the tree surface. By combining these signals through a sensor fusion algorithm, the system estimates the robot’s position, velocity, orientation, and limb dynamics in the body frame.

Visual sensing is also critical. The research by Carvalho et al. [42] showed that Pteromyini have visual pigments that are sensitive to short wavelengths, which enables them to maintain effective visual perception in low-light conditions. Inspired by this biological adaptation, we implement a sensing scheme that combines depth imaging with infrared thermal sensing to ensure reliable operation across a range of lighting and environmental conditions. The depth camera reconstructs 3D geometry by computing disparities between stereo image pairs, enabling the system to detect nearby trees, obstacles, and landing areas. The infrared camera supplements the system by capturing thermal radiation in the infrared spectrum, ensuring basic visual perception in dim or nighttime environments. Additionally, it can detect biological heat sources, facilitating non-invasive wildlife monitoring and the identification of habitat activity patterns, thereby adding a new dimension to ecological data acquisition. By employing advanced deep learning algorithms to process and fuse these multimodal sensing data, the system gains higher-level environmental understanding and task adaptation capabilities [43]. This enables the robot to more intelligently detect and track specific targets in forested areas as well as to reconstruct complex 3D environmental scenes.

### 2.2. Design and Architecture of PteroBot

**Figure 2:**
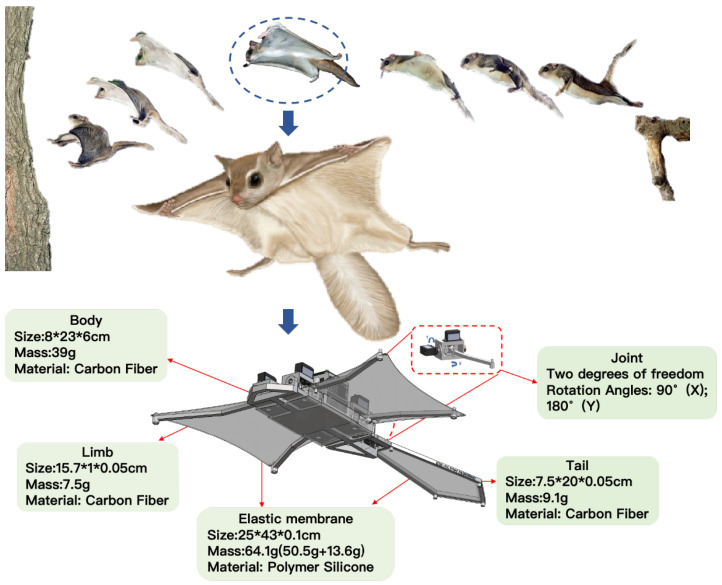
The design framework of PteroBot.

To realize the “climb–perch–glide” forest exploration paradigm, we designed and implemented a novel bioinspired robotic platform named PteroBot with a structural and functional design that draws inspiration from the morphology and gliding mechanism of Pteromyini. As shown in Figure 2, PteroBot consists of five primary components: the main body, forelimbs, hindlimbs, tail, and flexible wing membrane. The design emphasizes lightweight construction, aerodynamic performance, and multimodal locomotion capabilities.

The main body is fabricated from carbon fiber plates and optimized with a hollowed structure to minimize weight while maintaining overall stiffness. The onboard electronics, battery, and drive modules are centrally integrated within the torso, which conserves internal space and strategically positions the system’s center of mass ahead of the aerodynamic center, thereby enhancing gliding stability.

The forelimbs and hindlimbs of Pteromyini are each connected to the body via a two-degrees-of-freedom joint, allowing flexible movement in both vertical and horizontal directions. This configuration enables effective control during gliding and supports crawling functionality. The tail emulates Pteromyini’s form, with a trapezoidal configuration narrowing at the top and widening at the base. Constructed of a carbon fiber skeleton enveloped in a silicone membrane, it balances strength with flexibility. This component chiefly governs the robot’s pitch, promoting stable gliding and smooth landings.

The wing membrane is made of a lightweight and flexible silicone material with a Shore hardness of 10C, a tensile strength of 362 psi, and an elongation at break of up to 600%. Its material properties closely resemble the patagium of Pteromyini, providing excellent aerodynamic performance and durability. The membrane’s design ensures high aerodynamic efficiency and flight stability during gliding.

The design achieves a balance between structural complexity and mass efficiency, functional richness, and control simplicity. PteroBot is capable of executing cyclic “vertical climbing–static observation–aerial gliding” missions in complex forest environments. Its structure not only functionally replicates the biomechanics of Pteromyini but also provides a robust foundation for long-duration, low-disturbance, and high-mobility forest exploration.

### 2.3. Aerodynamic Characteristics and Control of Gliding

#### 2.3.1. Aerodynamic Characteristics of PteroBot

PteroBot utilizes a flexible membrane structure as its primary lift-generating surface during gliding. Studies in [35] based on simulation and wind tunnel experiments suggest that the aerodynamic variations induced by passive deformation of the membrane have a negligible effect on overall lift and drag during steady-state glide. Therefore, to simplify analysis and enhance the tractability of the aerodynamic model, the wing and tail surfaces are approximated as flat, rigid, and uniform structures in this study.

The structural framework of PteroBot consists of forelimbs, hindlimbs, and a central body, which can be abstracted as a rigid linkage system with a fixed topological configuration. During gliding, PteroBot actively modulates limb angles and tail posture to alter its mass distribution and aerodynamic attitude, thereby enabling dynamic adjustment of its aerodynamic parameters and real-time control over its flight trajectory and orientation.

The aerodynamic behavior of the PteroBot gliding model is governed by the lift coefficient ($C_{L}$) and drag coefficient ($C_{D}$), defined as follows:

(1) $$\left\{\begin{array}{c} C_{D}=\frac{2F_{D}}{\rho V^{2}S}\\ C_{L}=\frac{2F_{L}}{\rho V^{2}S} \end{array}\right.$$

where ρ and *V* represent the air density and velocity, respectively, while *S* denotes the area of the wing surface.

During gliding, the aerodynamic characteristics of the model are primarily influenced by the angle of attack (α). To quantitatively analyze the variation in lift and drag as a function of α, we performed a series of Computational Fluid Dynamics (CFD) simulations on the PteroBot gliding model. The simulations employed the *k*-*ε* turbulence model based on the Reynolds-Averaged Navier–Stokes (RANS) equations, which is well-suited for modeling boundary layer behavior and wake structures under the low Reynolds number flight conditions typical of PteroBot. The fluid medium was set as air with a density of $\rho =1.29\;\mathrm{kg}/m^{3}$, and the simulations were conducted under standard atmospheric pressure.

**Figure 3:**
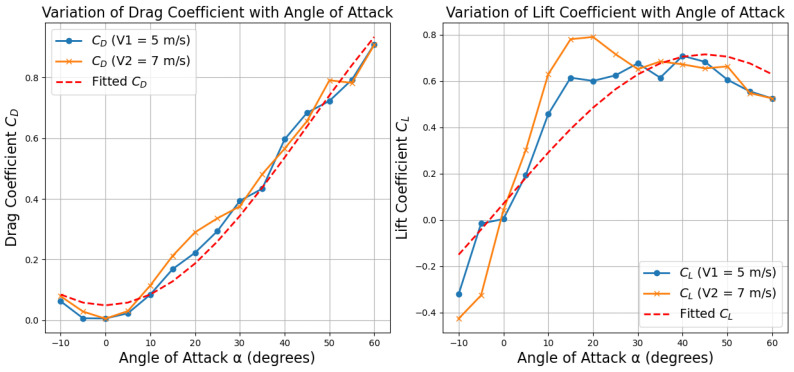
Variation of drag coefficient and lift coefficient with angle of attack.

According to findings in [30], the typical gliding speed of Pteromyini in natural environments ranges between 5 m/s and 7 m/s. Based on this, we evaluated the aerodynamic characteristics of the robotic model under airflow velocities of 5 m/s and 7 m/s in simulation. The angle of attack was varied from −10° to 60° in 5-degree increments, with aerodynamic performance analyzed for each configuration. At each configuration, the total aerodynamic lift and drag forces acting on the model were recorded. Using Equation (1), the lift coefficient ($C_{L}$) and drag coefficient ($C_{D}$) were computed as functions of α, as shown in Figure 3.

Simulation results indicated that the gliding model generates substantial aerodynamic force in the vertical direction at approximately α=40°. This posture aligns closely with the observed gliding behavior of Pteromyini, which adopts similar angles to achieve controlled descent and maneuverability.

Following the research in [44], a trigonometric fitting method was applied to model the relationship between aerodynamic coefficients and angle of attack. The resulting expressions are provided as follows:

(2) $$\left\{\begin{array}{c} C_{D}=4.91\times 10^{-2}+1.18\;\cdot \;\sin^{2}\left(\alpha \right)\\ C_{L}=6.97\times 10^{-2}+1.29\;\cdot \;\sin\left(\alpha \right)\cos\left(\alpha \right) \end{array}\right.$$

By Equation (2), the aerodynamic forces acting on the main wing membrane and the tail during gliding can be expressed as follows:

(3) $$\left\{\begin{array}{c} Fw=\frac{1}{2}\rho V^{2}S_{w}\left[\begin{array}{c} -C_{Dw}\\ 0\\ -C_{Lw} \end{array}\right]\\ Ft=\frac{1}{2}\rho V^{2}S_{t}\left[\begin{array}{c} -C_{Dt}\\ 0\\ -C_{Lt} \end{array}\right] \end{array}\right.$$

where $F_{w}$ and $F_{t}$ denote the aerodynamic force vectors acting on the wing membrane and the tail, respectively, ρ is the air density, *V* is the relative airspeed of the robot, $S_{w}$ and $S_{t}$ are the respective surface areas of the wing membrane and tail, and $C_{Dw},C_{Lw},C_{Dt},C_{Lt}$ are the corresponding aerodynamic drag and lift coefficients.

**Figure 4:**
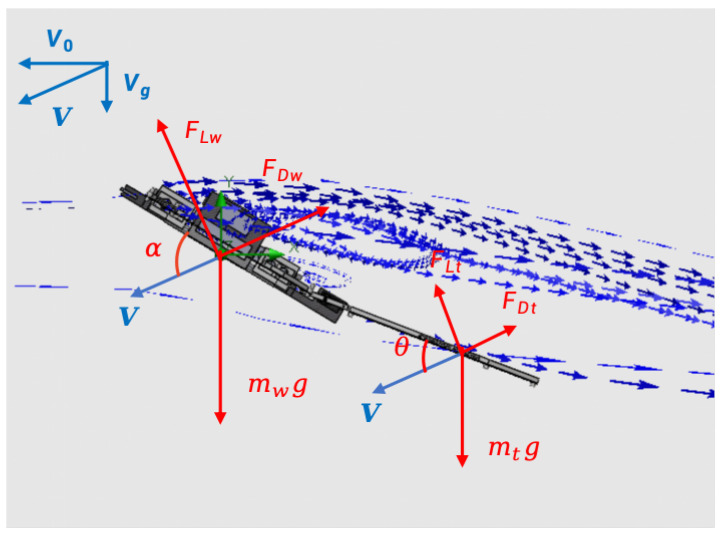
Aerodynamic analysis of PteroBot during gliding.

Figure 4 presents aerodynamic analysis of PteroBot during gliding. We employ a Newton–Euler formulation to analyze the gliding dynamics of the robot model. The translational motion in the inertial frame is governed by the following equations:

(4) $$\left\{\begin{array}{c} m\ddot{X}=mg+R\left(F_{w}+F_{t}\right)\\ X={\left[x\;y\;z\right]}^{T} \end{array}\right.$$

where *m* is the mass of the robot, $\ddot{X}={\left[\ddot{x}\;\ddot{y}\;\ddot{z}\right]}^{T}$ is the acceleration of the robot’s Center of Mass (CoM), $g={\left[0\;0\;g\right]}^{T}$ is the gravitational acceleration, R is the rotation matrix representing the transformation between the robot body frame and the reference frame, and $F_{w}+F_{t}$ is the total aerodynamic force generated by the wing and tail surfaces.

The rotational dynamics of PteroBot about its center of mass follow rigid-body dynamics and can be described by the Euler angular momentum equation. Let I be the inertia tensor about the center of mass, $\omega ={\left[\omega_{x}\;\omega_{y}\;\omega_{z}\right]}^{T}$ the angular velocity vector, and τ the total aerodynamic torque acting on the body. The rotational dynamics are then provided by

(5) $$I\dot{\omega }+\omega \times \left(I\omega \right)=\tau$$

where $\dot{\omega }$ is the angular acceleration and the gyroscopic term ω×(Iω) captures inertial coupling effects in a rotating reference frame.

The aerodynamic torque τ arises from the moments generated by aerodynamic forces acting on the wing membrane and tail, expressed as follows:

(6) $$\tau =r_{w}\times F_{w}+r_{t}\times F_{t}$$

where $r_{w}$ and $r_{t}$ are the position vectors from the center of mass to the aerodynamic centers of the wing membrane and tail, respectively. To facilitate control system design, Euler angles (ϕ,θ,ψ) are introduced to describe the robot’s orientation. The relationship between the Euler angles and angular velocity is provided by the kinematic equation

(7) $$\dot{\eta }=T\left(\eta \right)\omega$$

where $\eta ={\left[\phi \;\theta \;\psi \right]}^{T}$ and T(η) is the transformation matrix that maps angular velocities to Euler angle rates.

#### 2.3.2. Gliding Attitude Controller

In related studies, advanced control strategies such as reinforcement learning [45] and robust compensation techniques [46] have been developed for real-time feedback control systems. These approaches have demonstrated strong performance in terms of stability and robustness. In this study, PteroBot is classified as a low aspect ratio (AR = 1.72) and low Reynolds number glider. Its gliding duration is short, and it requires a large angle of attack to generate sufficient lift. These characteristics demand high responsiveness and stability from the attitude control module. To address these requirements, a cascaded closed-loop controller is implemented. The outer loop governs attitude regulation, while the inner loop manages angular rate control. This control framework simplifies the complex dynamics associated with low-speed gliding and achieves a balance between robustness and real-time responsiveness.

**Figure 5:**
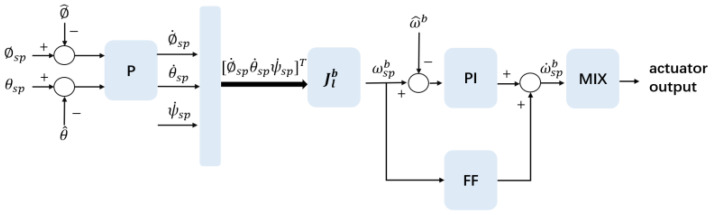
Cascaded closed-loop attitude controller. The physical meanings of the symbols are as follows. $\Psi_{sp}=\left[\phi_{sp},\theta_{sp},\psi_{sp}\right]$: Target attitude, including setpoints for roll angle ($\phi_{sp}$), pitch angle ($\theta_{sp}$), and yaw angle ($\psi_{sp}$). $\hat{\Psi }=\left[\hat{\phi },\hat{\theta },\hat{\psi }\right]$: Estimated current attitude measured in real time by sensors, including roll, pitch, and yaw angles. *P*: Proportional controller module, which calculates the attitude error and generates the target angular velocity. $\omega_{sp}^{b}$: Target angular velocity, the output of the proportional controller, guiding the inner control loop. $J_{l}^{b}$: Transformation matrix, converting the target angular velocity from the local coordinate system to the body coordinate system. ${\hat{\omega }}^{b}$: Current angular velocity, measured by sensors such as gyroscopes. PI: Proportional-integral controller module, which generates the target angular acceleration based on angular velocity error. FF: Feedforward module, which directly generates compensation signals to mitigate the effect of aerodynamic damping. MIX: Mixer module, which allocates the target angular accelerations to specific actuators and generates control signals. δ: Actuator deflection, representing the specific control commands for servos or other actuators.

As shown in Figure 5, the attitude controller (outer loop) first calculates the error between the current attitude and the target attitude, applying proportional gain to generate a target angular velocity. The inner loop’s rate controller then receives this target angular velocity and compares it with the actual angular velocity provided by IMU. The error signal is calculated; due to the limited time window available for attitude adjustments during gliding, the robot requires high maneuverability. To address this, the inner loop controller combines a feedforward module with a Proportional-Integral (PI) module to generate angular acceleration signals that compensate for aerodynamic damping, ensuring a quick response to external disturbances.

The attitude correction signal from the outer loop and the angular acceleration signal from the inner loop are then passed to a data mixer module. The mixing controller module is designed to map the robot’s pitch, yaw, and roll attitude changes to the actuators. During gliding, PteroBot has five degrees of freedom to control its posture, including the limbs and tail.

Pitch control is achieved by actuating the tail in the sagittal plane. By rotating the tail up or down, the robot shifts the mass distribution relative to the center of mass, effectively altering the pitch moment of inertia $I_{yy}$. In the absence of external torques, the conservation of angular momentum dictates that

(8) $$\frac{d}{dt}\left(I_{yy}\;\omega_{\mathrm{pitch}}\right)=0\;\Rightarrow \;\Delta I_{yy}\;\cdot \;\omega_{\mathrm{pitch}}+I_{yy}\;\cdot \;\Delta \omega_{\mathrm{pitch}}=0$$

This implies that tail deflection causes a reactive change in pitch rate $\omega_{\mathrm{pitch}}$, allowing the robot to adjust its nose-up or nose-down attitude. The control law for tail movement is provided by

(9) $$\delta_{\mathrm{tail}}=k_{p}\;u_{\mathrm{pitch}}$$

where $k_{p}$ is a gain factor that encodes the dynamic coupling between tail deflection and pitch rate change, considering mass distribution and inertial properties.

For yaw control, the Pteromyini achieves directional changes by lifting one side’s forelimb in combination with the tail. To reduce the coupling between yaw and pitch controls, we improved the system by using antagonistic deflection of diagonal limb pairs to control yaw, such as lifting the left forelimb and right rear limb; this generates differential drag forces through the opposite limb pairs while maintaining longitudinal balance:

(10) $$\left\{\begin{array}{c} \delta_{\mathrm{LF}}=-\delta_{\mathrm{RR}}=k_{y}u_{\mathrm{yaw}}\\ \delta_{\mathrm{RF}}=-\delta_{\mathrm{LR}}=-k_{y}u_{\mathrm{yaw}}\\ k_{y}=\frac{b}{2V}\;\cdot \;\frac{\partial C_{n}}{\partial \delta }\;\cdot \;\frac{\rho V^{2}S}{2I_{zz}} \end{array}\right.$$

where *b* represents the wingspan, $I_{zz}$ is the yaw moment of inertia, and $\partial C_{n}/\partial \delta$ is the derivative of the yaw moment coefficient.

Roll control is achieved through the coordinated movement of ipsilateral limb pairs, shifting the lateral Center of Gravity (CoG) to induce bank angles. The coordinated deflection of left or right limb pairs creates asymmetric mass distribution:

(11) $$\left\{\begin{array}{c} \delta_{\mathrm{LF}}=\delta_{\mathrm{LR}}=k_{r}u_{\mathrm{roll}}\\ \delta_{\mathrm{RF}}=\delta_{\mathrm{RR}}=-k_{r}u_{\mathrm{roll}}\\ k_{r}=\frac{mgd}{I_{xx}}\;\cdot \;\frac{\partial \phi }{\partial \delta } \end{array}\right.$$

where *d* is the effective moment arm between the CoG and the limb attachments, *m* is the robot’s mass, and $I_{xx}$ is the roll moment of inertia. The roll authority coefficient $k_{r}$ ensures a proportional relationship between limb deflection and angular acceleration.

After integrating these mechanisms, the mixed control matrix for the Pteromyini’s attitude controller is derived as shown below.

(12) $$\left[\begin{array}{c} \delta_{\mathrm{tail}}\\ \delta_{\mathrm{LF}}\\ \delta_{\mathrm{RF}}\\ \delta_{\mathrm{LR}}\\ \delta_{\mathrm{RR}} \end{array}\right]=\left[\begin{array}{ccc} k_{p} & 0 & 0\\ 0 & -k_{y} & k_{r}\\ 0 & k_{y} & -k_{r}\\ 0 & k_{y} & k_{r}\\ 0 & -k_{y} & -k_{r} \end{array}\right]\left[\begin{array}{c} u_{\mathrm{pitch}}\\ u_{\mathrm{yaw}}\\ u_{\mathrm{roll}} \end{array}\right]$$

This mixed control matrix directly maps the control commands to specific servo movements, allowing for real-time adjustments of the center of gravity and overall aerodynamic force distribution of the wing. By redistributing lift and drag, the robot can maintain stable gliding even in complex airflow environments. The entire process forms a closed-loop system, continuously collecting sensor data that are fed back to the control module for dynamic adjustments and optimization.

## 3. Results

### 3.1. Simulation of Gliding Attitude Control

#### 3.1.1. Simulation of Gliding Control Strategy

To evaluate the optimal gliding performance of the PteroBot model and validate the effectiveness of its attitude controller, we conducted a series of simulation experiments based on the established gliding dynamics framework. Observational studies by Paskins et al. [47] indicate that Pteromyini typically maintains an angle of attack near $40^{\circ }$ during sustained gliding to achieve long-range travel. As it approaches landing, the body gradually increases its pitch angle, reaching nearly $90^{\circ }$ to reduce descent velocity and enhance landing stability.

Inspired by this biological gliding strategy, we adopted a similar glide-phase control scheme for simulation. The robot was initialized at a glide height of 2 m with a horizontal velocity of 5 m/s. During gliding, the attitude controller adjusts and maintains the glide angle at a preset value. When the altitude drops below 0.5 m, the robot enters a landing preparation phase, increasing the pitch angle to $75^{\circ }$ to initiate deceleration and stabilize its descent.

The simulation assumed the following parameters: total mass of 1.2 kg, wing membrane area of 0.12 m^2^, tail surface area of 0.014 m^2^, air density $\rho =1.29\;kg/m^{3}$, and gravitational acceleration $g=9.81\;m/s^{2}$. By substituting the above assumptions into Equations (2) and (3), the aerodynamic forces acting on the model under simulation conditions can be calculated. Using the Newton–Euler dynamic framework described in Equations (4) and (5), the translational acceleration and attitude states at each time step are then determined. The controller was implemented in Simulink to perform real-time state updates. The simulation time step was set to 0.01 s, and the simulation terminated when the altitude dropped below 0.

**Figure 6:**
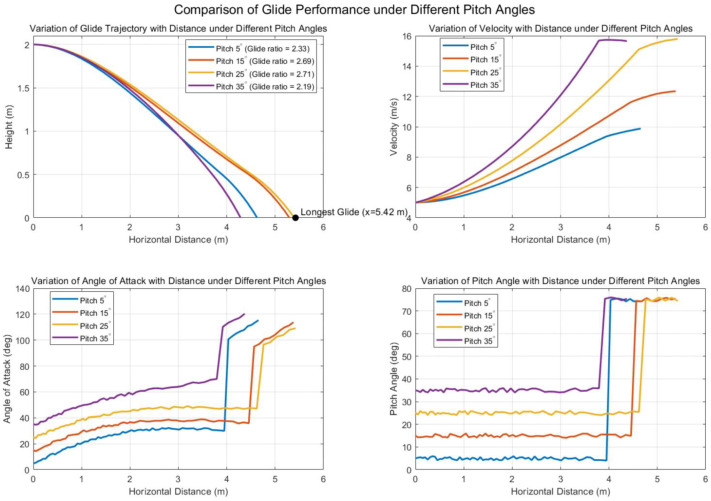
Comparison of glide performance under different pitch angles.

We simulated gliding performance under four pitch angles: $5^{\circ }$, $15^{\circ }$, $25^{\circ }$, and $35^{\circ }$. The results are shown in Figure 6. The data indicate that the robot achieves its best gliding performance when the pitch angle is within the range of $15^{\circ }$ to $25^{\circ }$, with a peak glide ratio of 2.7. Within this range, the angle of attack remains consistently near $40^{\circ }$, aligning well with observed Pteromyini gliding behavior. As the robot descends to 0.5 m, increasing the pitch angle leads to a significant reduction in velocity, confirming the effectiveness of the deceleration strategy.

The pitch angle trajectory further demonstrates that the attitude controller exhibits fast and stable response, achieving rapid convergence to the target angle without overshoot or oscillation. Throughout the gliding phase, the center-of-mass trajectory remains smooth and the attitude transitions are continuous and stable, with no evidence of high-frequency oscillations or divergence. These results validate the reliability and practicality of the proposed gliding dynamics model and control framework.

#### 3.1.2. Simulation of Turning Control Strategy

Achieving a high glide ratio alone is not sufficient to ensure effective aerial maneuverability. The gliding model must also possess adequate agility to enable precise trajectory control and safe arrival at target locations. In gliding flight, directional changes are typically induced by asymmetric aerodynamic forces generated along specific body axes. By modulating limb configurations to alter its aerodynamic posture, PteroBot can produce lateral force components, enabling controllable directional adjustments during flight.

To assess this capability, we conducted turning simulations based on the previously established aerodynamic model and Newton–Euler dynamic framework. The goal was to evaluate the feasibility and effectiveness of heading control through active attitude modulation, supporting future development of path planning and navigation strategies.

The simulations were carried out using the same physical parameters and initial conditions as described in the previous section. A right-handed inertial coordinate frame was defined and the robot was initialized at position (x,y,z)=(0,0,2)m, gliding along the positive x-axis at a velocity of 5 m/s. Following the results from Section 3.1, the pitch angle was maintained at $25^{\circ }$ throughout the glide to ensure balanced lift and drag. Upon receiving a turning command, the controller adjusted the roll angle to a predefined value. This attitude change caused the wing’s surface normal to deviate from the incoming airflow direction, thereby generating a lateral aerodynamic force component. According to aerodynamic analysis, this lateral force locally disrupts the symmetry of the lift distribution and produces a yaw moment that shifts the robot’s heading direction.

The aerodynamic model indicates that increasing the roll angle amplifies the lateral force, enhancing yaw maneuverability. However, excessive roll may compromise aerodynamic stability during gliding, increasing the risk of instability and uncontrolled rolling. To balance maneuverability and flight stability, we set roll angles as $45^{\circ }$, $55^{\circ }$, and $65^{\circ }$ to evaluate gliding performance and maneuverability under varying control intensities. The simulation terminated when the robot reached ground level (z=0m).

**Figure 7:**
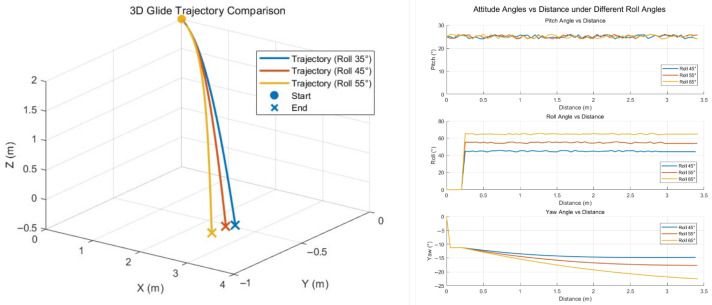
Gliding trajectory and attitude angle changes in turning simulation.

The simulation results are shown in Figure 7, presenting the 3D gliding trajectories of PteroBot under each roll condition. The trajectories clearly demonstrate that roll-induced attitude adjustments produce significant deviations in flight direction, resulting in noticeable horizontal turning. Compared to the baseline straight-line glide, the curved trajectories confirm the role of roll-induced lateral aerodynamic forces in enabling yaw maneuvering.

Among the three test cases, an increase in roll angle corresponded with larger trajectory deflections and higher final yaw angles. These results confirm that greater roll inputs generate stronger lateral forces, leading to enhanced maneuverability. The observed relationship between roll angle and final heading displacement verifies the tunability of yaw control via roll modulation. Additionally, attitude angle changes during simulation show that the controller responded rapidly to the turning command, achieving the desired roll angle within a short time. The pitch angle remained within the target range throughout the turning maneuver, indicating effective decoupling and stability of the control system.

However, the simulations were conducted under idealized disturbance-free conditions, without accounting for wind perturbations, structural vibrations, or external stochastic disturbances. As a result, no loss of control was observed at high roll angles. Further physical experiments in realistic outdoor environments are needed to evaluate the limits of directional stability and to investigate potential aerodynamic imbalances induced by extreme roll maneuvers.

### 3.2. Experimental Validation of Exploration and Perception Capabilities in Forest Environments

#### 3.2.1. Experiment of Locomotion Capabilities

**Figure 8:**
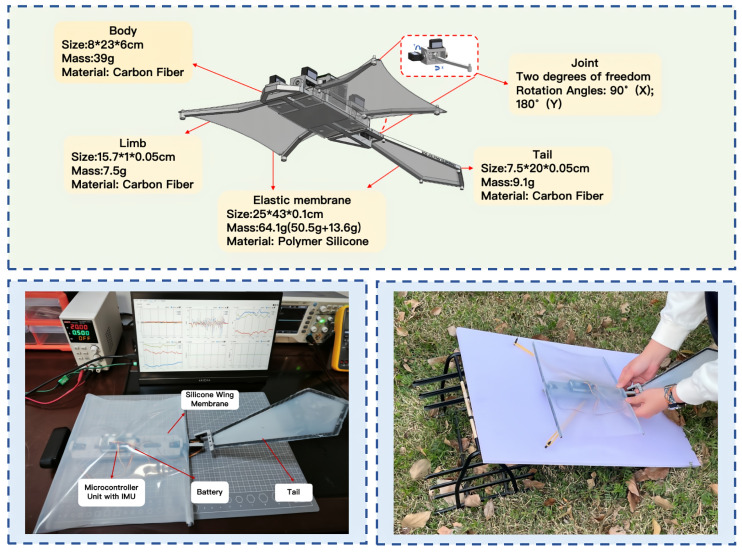
Simplified glider model for experiment.

To further validate the feasibility and stability of PteroBot’s gliding attitude control in real-world conditions, a simplified experimental glider model was developed based on the original prototype design, as shown in Figure 8. This test platform preserves the five essential degrees of freedom required for gliding: two actuated joints in the forelimbs, two in the hindlimbs, and one in the tail. It is also equipped with key onboard modules, including an Inertial Measurement Unit (IMU) and a Microcontroller Unit (MCU) to support attitude sensing and control. Compared to the full-scale system, the test model has a lighter structure and a more compact layout, making it well suited for medium- and low-altitude glide testing as well as attitude response evaluation.

Experiments were conducted in outdoor conditions with mild natural wind, where wind speeds ranged from 0.5 m/s to 1.2 m/s. The initial parameters used in the experiment matched those in the simulation. A custom-built catapult was employed to launch the model with a consistent initial velocity. The launch pitch angle was set to $45^{\circ }$. After release, the glider followed an oblique ballistic trajectory. When the vertical velocity decreased to zero, indicating the apex of flight, the system automatically activated the attitude control algorithm to initiate the gliding phase and begin active aerodynamic adjustments.

**Figure 9:**
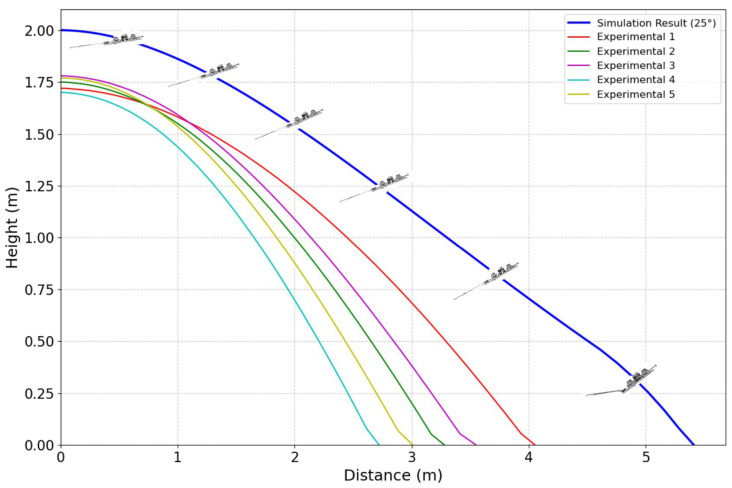
Results of controlled gliding experiments.

During the gliding experiments, the robot utilized the onboard JY901B Inertial Measurement Unit (IMU) (WitMotion, Shenzhen, China) to capture tri-axial acceleration, angular velocity, and magnetic field data at a sampling frequency of 200 Hz. Real-time attitude angles were estimated by integrating angular velocity, while displacement was derived from integrated acceleration. However, direct integration often leads to significant errors due to sensor noise, drift, and accumulated integration bias. To address this, a dynamic Kalman filter was applied to process the IMU data, yielding more accurate pose estimations in dynamic environments. The attitude estimation achieved a precision of 0.2 degrees, while displacement estimation reached an accuracy of 0.01 m. The attitude control strategy used in the experiment was consistent with that employed in simulation. Based on feedback from the IMU, the controller was able to adjust the robot’s orientation to match target attitude values. A total of five experimental trials were conducted, and the results are shown in Figure 9. The average glide ratio across the five trials was 2.02, which is slightly lower than the simulated glide ratio of 2.71. This discrepancy can be attributed to several factors. First, the real-world environment presents low-speed airflow disturbances and ground effect phenomena, which are difficult to accurately replicate in simulation. These factors can impact the stability and symmetry of the aerodynamic forces. Second, the wing membrane on the physical prototype is made of flexible material which exhibited elastic oscillations during flight, potentially reducing lift generation efficiency. Additionally, the actuation system experienced response delays and control inaccuracies, which may have reduced the responsiveness of the attitude controller and consequently degraded gliding performance.

Despite the slightly lower glide ratio compared to simulation, the experimental glide trajectories remained smooth and the attitude adjustments were executed quickly and stably. These results confirm the feasibility and effectiveness of the proposed attitude control strategy in the physical system. The observed glide ratio remained within an acceptable range, demonstrating that the robot possesses adequate gliding capability to support short-range aerial mobility in forested environments.

In addition to the gliding tests, we further evaluated PteroBot’s turning control capabilities. Preliminary trials with varying roll angles indicated that the robot was prone to aerodynamic imbalance when the roll angle exceeded $45^{\circ }$, leading to instability and loss of attitude control. To ensure safety and experimental consistency, the maximum roll angle for the formal trials was limited to $40^{\circ }$.

Under this constraint, a total of ten turning experiments were conducted. Trials 1 through 5 applied roll inputs for leftward turning, while Trials 6 through 10 were configured for rightward turning. During each flight, the Inertial Measurement Unit (IMU) was used to estimate the trajectory and measure the lateral deviation as an indicator of yaw control performance.

**Figure 10:**
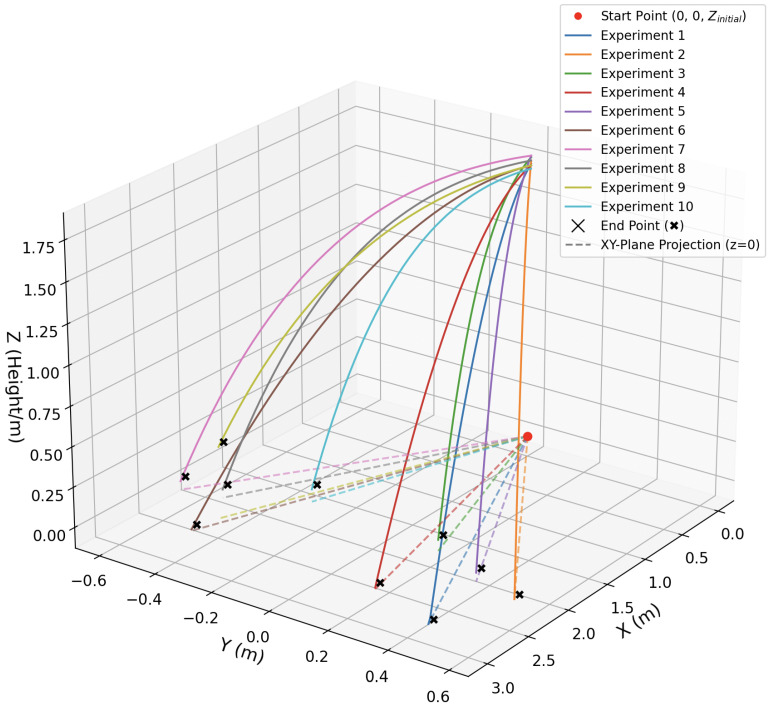
Results of controlled turning experiments.

The results are shown in Figure 10. For leftward turns, the average lateral deviation was 0.39 m, with a maximum recorded deviation of 0.58 m. For rightward turns, the average deviation was 0.40 m, with a maximum of 0.61 m. The results demonstrate both effective directional control and consistent symmetry between left and right turns. No significant instability or loss of glide was observed within the defined roll angle limit. These findings confirm the practical feasibility of the roll-induced turning strategy in a physical system, enabling meaningful directional adjustments while maintaining gliding stability. Such capabilities are essential for tasks such as path correction and target following in forest exploration scenarios.

Although the maximum deviation is inherently constrained by the roll angle limit, the method achieves effective maneuverability with minimal structural complexity and low control overhead. These experiments provide performance benchmarks and empirical evidence that can support future developments in glide path planning and turning control strategies.

#### 3.2.2. Energy Consumption and Acoustic Disturbance Performance of PteroBot

To evaluate PteroBot’s suitability for ecologically sensitive forest exploration tasks such as long-duration wildlife monitoring and environmental conservation, a series of experiments were conducted to assess its system-level energy efficiency and acoustic impact. For benchmarking, we selected a commercially available multirotor drone, the DJI Phantom 4, which is widely used in similar applications.

We evaluated the power consumption of the PteroBot system under three operational conditions: gliding, perching, and peak load. PteroBot is powered by a 2600 mAh lithium-polymer battery with a nominal voltage of 12 V. The average power draw of the sensor modules is approximately 3 W, while the Raspberry Pi (Raspberry Pi Foundation, Cambridge, UK) controller consumes around 2 W. Additional components, including communication modules, contribute an estimated 1.5 W to the total system power. Under typical operating conditions with a 20% to 50% load, each servo draws an average current of approximately 400 mA.

**Figure 11:**
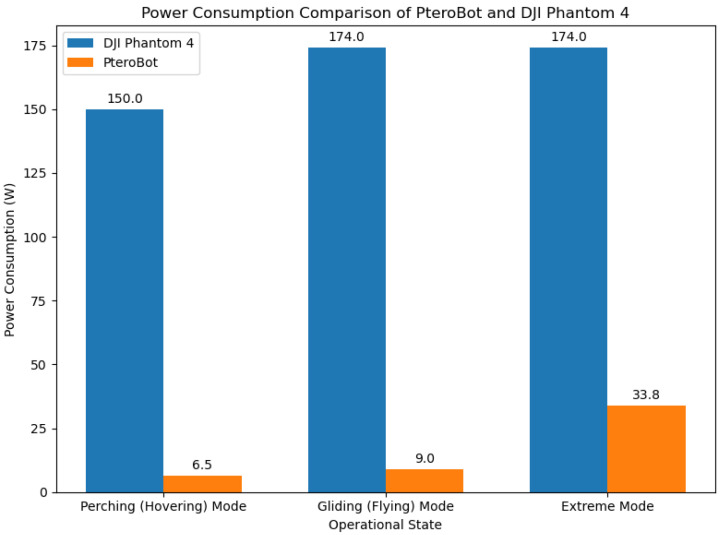
Power consumption comparison of PteroBot and DJI Phantom 4.

As shown in Figure 11, when all joints are actuated simultaneously, the system reaches a peak power consumption of 33.8 W. During controlled gliding, only a subset of servos is intermittently activated for posture adjustment, resulting in a power draw of 9 W. In perch mode, where actuation is minimal and only the sensors and main controller are active, power consumption drops to 6.5 W. For comparison, we tested the DJI Phantom 4 drone (DJI, Shenzhen, China). Its estimated flight power consumption based on battery capacity and endurance time is approximately 174 W. In passive gliding conditions, its power draw remains as high as 150 W.

Assuming a typical forest exploration mission that involves a perching (hovering) to gliding (flying) time ratio of 5:1, PteroBot achieves approximately 40 times greater endurance than the Phantom 4 despite operating on only half its battery capacity. This advantage makes PteroBot particularly well-suited for long-duration missions such as routine forest fire inspections or military reconnaissance operations.

**Figure 12:**
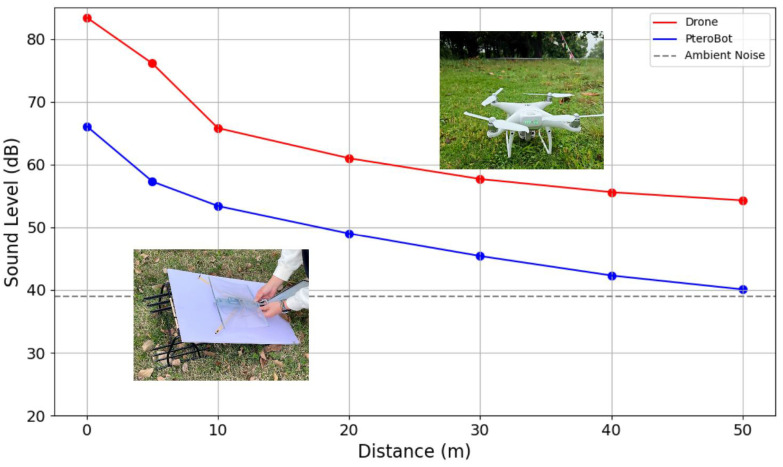
Drone and PteroBot noise levels across distances.

Moreover, we measured the sound pressure levels generated by PteroBot and the DJI Phantom 4 during operation. A DL333211 sound level meter with 0.1 dB resolution was used (Deli Group Co., Ltd., Ningbo, China). Measurements were conducted in a semi-quiet forest setting with an ambient noise level of 39 dB. The results are shown in Figure 12.

The Phantom 4 exhibited a peak noise level of 83.4 dB during working. Even at 50 m, its noise level remained at 54.3 dB, surpassing the IUCN-recommended 10 dB above ambient threshold for preserving natural acoustic environments. In contrast, PteroBot does not rely on high-speed rotors. Its acoustic output originates from intermittent actuator motion and low-speed airflow during gliding. At a distance of 10 m, it produces less than 55 dB, and this attenuates rapidly with distance; beyond 15 m, the noise level closely approaches the ambient baseline.

Research in [48] has indicated that continuous noise above 55 dB can induce physiological stress in wildlife, leading to foraging disruption and avoidance behavior, thereby interfering with normal ecological activity. This indicates that PteroBot offers superior acoustic stealth and minimal ecological disruption, being better suited for long-term monitoring, wildlife observation, and conservation tasks in environmentally sensitive areas.

**Figure 13:**
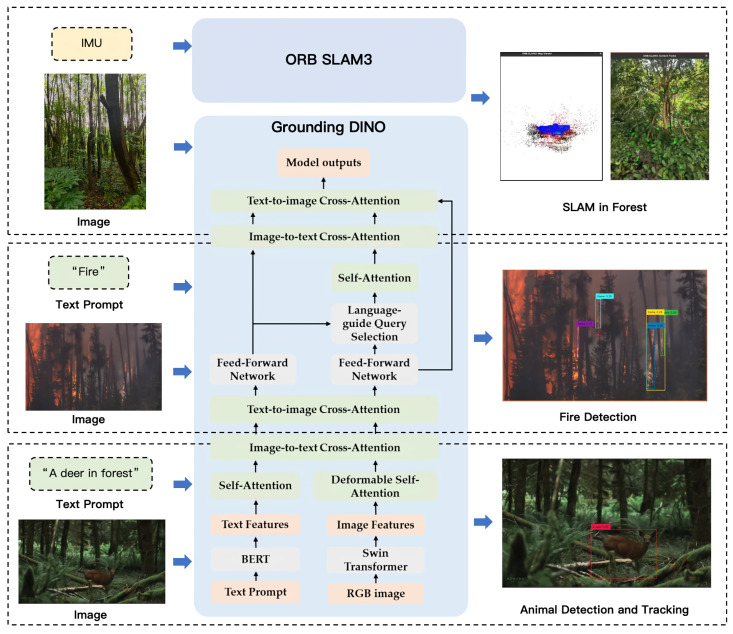
Common forest monitoring task tests.

To demonstrate PteroBot’s advantages in forest exploration tasks such as routine wildfire inspection and wildlife monitoring and conservation, we deployed the ORB-SLAM3 system [49] and the multimodal open-set detection model Grounding DINO [50] to enable task-specific target identification, as shown in Figure 13. Both the detection score threshold and text-matching threshold were set to 0.25. In the wildlife monitoring and conservation scenario, the text prompt was set to “a deer in the forest”. The system successfully identified a deer walking among the trees with a confidence score of 0.92. In the wildfire inspection scenario, the text prompt was set to “fire” and the system accurately detected a flame region in the image, achieving a confidence score of 0.83.

These experiments demonstrate that PteroBot offers advantages such as low energy consumption and minimal environmental disturbance thanks to its bioinspired structural design and gliding-based locomotion. These characteristics address limitations of rotorcraft UAVs such as short flight endurance and high acoustic noise. As a result, PteroBot presents a novel and ecologically compatible solution for long-duration forest exploration tasks, particularly in scenarios such as continuous environmental monitoring and wildlife observation and conservation.

## 4. Discussion

### 4.1. Summary and Discussion

This study introduces PteroBot, a bioinspired gliding robot for forest exploration. The proposed robotic system encompasses a bioinspired structural design, aerodynamic modeling and analysis, and attitude controller development. PteroBot demonstrates strong performance in both gliding and turning, as validated through simulation and physical testing. To further clarify the contributions of this work, the key innovations are summarized as follows:

1. Bioinspired forest exploration paradigm: PteroBot adopts a forest exploration strategy inspired by Pteromyini’s “climb–perch–glide” locomotion, marking the first application of biological gliding mechanisms to contact-based forest exploration. Unlike wheeled or legged robots constrained by terrain, PteroBot leverages passive gliding to avoid direct contact with complex ground features and obstacles, making more efficient use of the surrounding environment for mobility and sensing.
2. Cascaded closed-loop attitude control architecture: The integration of a cascaded attitude and rate controller enables responsive and stable posture regulation under the low Reynolds number and high angle-of-attack conditions of forest gliding. Compared to reinforcement learning or prescribed performance control approaches that require extensive training or complex parameterization, our method balances simplicity and real-time robustness for gliding attitude control.
3. Enhanced gliding performance and maneuverability: Current bioinspired gliders primarily focus on pitch adjustment for attitude control, with limited implementation of steering capabilities. In this study, inspired by the gliding posture modulation of Pteromyini, we achieve controlled turning via a dedicated attitude controller. Experimental results show that the minimum turning radius reaches 5.7 m under balanced initial conditions, with symmetrical performance in both left and right turning directions. Given that the average tree spacing in forest environments is approximately 6–8 m, this maneuverability is sufficient for navigating between trees. Furthermore, PteroBot attains a maximum glide ratio of 2.02, representing an 11.4% improvement over existing bioinspired gliders and approaching the gliding efficiency observed in real flying squirrels.
4. Low energy consumption and minimal ecological disturbance: PteroBot’s bioinspired structural design and gliding-based locomotion enable significantly lower energy consumption and reduced environmental impact. Unlike rotorcraft UAVs, which suffer from limited endurance and generate high acoustic noise, PteroBot operates with passive aerodynamics and intermittent actuation, thereby avoiding continuous power draw and mechanical disturbance. These characteristics offer a novel solution for long-duration forest exploration tasks, particularly in applications such as persistent environmental monitoring and wildlife observation and conservation.

### 4.2. Limitations and Challenges

While this study demonstrates the preliminary feasibility of PteroBot’s performing “climb–perch–glide” exploration tasks in forest environments, several challenges remain in both system design and field deployment. These challenges fall mainly into two categories: physical constraints related to size and weight, and architectural limitations concerning onboard computation and inference capabilities.

#### 4.2.1. Size and Weight Constraints

PteroBot needs to support multiple locomotion modes, including gliding, climbing, and jumping. These functional requirements increase mechanical complexity and necessitate several actuated degrees of freedom, contributing significantly to system weight. In addition, forest monitoring missions often require specific sensor payloads such as thermal imagers, hyperspectral cameras, or gas detectors. These modules add further weight and volume.

To achieve sufficient lift during gliding, the robot must maintain a minimum wing area. This aerodynamic requirement also imposes a lower bound on the system’s physical size. The current experimental model has a wingspan of 25 × 35 cm and a total weight of 485.6 g, achieving a payload ratio of 63.3%. The full prototype, which integrates climbing and takeoff components, reaches a mass of 1.2 kg and extends to 50 × 70 cm in size. These specifications are adequate for typical forest tasks; however, further reduction in size and mass would improve stealth, reduce energy consumption, and increase operational endurance.

Motors currently account for approximately 46.6% of the total system weight. Reducing this proportion is essential for further optimization. Future designs should explore compact and lightweight actuators with high torque density.

#### 4.2.2. Computational Constraints

Another key challenge is the limited onboard computational capacity. The current system offloads most perception and inference tasks to a remote cloud server. These tasks include visual recognition, object detection, and Simultaneous Localization and Mapping (SLAM). While this architecture provides strong computational power, it also introduces latency and relies heavily on network stability.

In densely forested or mountainous environments, wireless communication may become unreliable or unavailable. These conditions can significantly impact the system’s real-time responsiveness and task safety. A more robust approach would be to deploy all perception and decision-making models locally. This would improve autonomy, reduce latency, and make the system more resilient to communication failures.

However, implementing local inference introduces two major challenges. First, the embedded computing platform must provide sufficient processing power while remaining compact and energy-efficient. This requirement is constrained by both payload capacity and battery limitations. Second, the perception models themselves must be lightweight and efficient. Reducing model complexity without compromising accuracy remains a technical challenge. Promising approaches include model pruning, quantization, and knowledge distillation. Integrating these techniques effectively into a deployable system continues to be an open research problem.

### 4.3. Future Works

Future research will focus on structural integration, control strategy enhancement, perception model optimization, and multi-agent collaboration, with the goal of improving the autonomy and adaptability of PteroBot in real-world forest environments.

At the structural level, efforts will be directed towards tighter integration of gliding and climbing mechanisms to improve spatial efficiency and coordination across locomotion modes. Use of lightweight materials, topology optimization, and modular design will support further reductions in size and mass while maintaining aerodynamic performance and structural robustness. On the control side, more advanced adaptive controllers will be developed to enable smooth transitions between climbing, perching, and gliding states. Integration of real-time sensor feedback with path planning algorithms will support autonomous navigation, obstacle avoidance, and decision-making in unstructured forest terrain. In terms of perception, the perception and control systems will be restructured to support lightweight multimodal inference. This will enable real-time onboard environmental understanding without reliance on external computation. Additionally, multi-robot collaboration will be explored through the implementation of swarm intelligence algorithms. This approach will allow multiple PteroBot to coordinate exploration, data sharing, and task allocation, improving the efficiency and coverage of forest exploration operations. Ultimately, these efforts aim to transform the Pteromyini robot into a scalable, intelligent, and collaborative platform capable of addressing critical challenges in forest exploration and conservation.

## 5. Conclusions

In this paper, we propose a bioinspired gliding robot called PteroBot designed for forest exploration and establish a system framework integrating multimodal perception, gliding dynamics modeling, and closed-loop attitude control. Experimental results confirm the aerodynamic effectiveness of the bioinspired structure, demonstrating stable gliding performance under the designed attitude controller and validating the roll-induced turning strategy. Compared to conventional aerial platforms such as UAVs, PteroBot offers significantly lower energy consumption and reduced ecological disturbance, making it particularly well suited for sustained forest exploration and tasks involving wildlife observation and conservation. This work demonstrates the potential of biologically-inspired environmentally compatible robots for agile and intelligent forest exploration. Through a combination of mechanical design, control innovation, and sensor integration, PteroBot represents a step toward practical and scalable solutions for ecological monitoring, conservation, and environmental science.

## Data Availability

The data and code of the current study can be obtained from the authors upon reasonable request.
